# Effectiveness of endotracheal intubation and mask ventilation procedural skills training on second-year student using modified Peyton’s Four-Step approach during COVID-19 pandemic

**DOI:** 10.1080/10872981.2023.2256540

**Published:** 2023-09-07

**Authors:** Aldy Heriwardito, Andi Ade Wijaya Ramlan, Abd. Basith, Lara Aristya

**Affiliations:** Department of Anesthesiology and Intensive Care, Cipto Mangunkusumo General Hospital, Faculty of Medicine University of Indonesia, DKI Jakarta, Indonesia

**Keywords:** Clinical skill, intubation, medical student, Peyton’s Four Step approach, online learning

## Abstract

**Background:**

Airway emergency is the reason behind competency in mask ventilation and intubation skills for doctors. Procedural skills are taught through face-to-face training. However, the COVID-19 pandemic has had an impact on the education system, including medical education. The face-to-face training method cannot be carried out and requires a modification to an online method. Therefore, Peyton’s Four-Step Approach is modified to adapt this change.

**Objective:**

Assessing the effectiveness of learning methods between modified Peyton’s Four-Step approach and classic Peyton’s Four Step approach in learning basic skills of mask ventilation and intubation during the COVID-19 pandemic.

**Method:**

This was an experimental study with two groups of subjects in the Basic ClinicalSkills module of endotracheal intubation and mask ventilation at the Faculty ofMedicine, Universitas Indonesia during February-June 2022. The first group received training with classic Peyton’s Four-Step approach and another group with modified Peyton’s Four-Step approach. Both groups were tested and assessed using rubric score and global rating score, and asked to fill a feedback questionnaire.

**Results:**

This study was conducted with 133 students in the classic group and 96 students in the modified group. The median rubric score was 21.2 for both groups and the global rating score showed 82%and 78% students passed, for classic and modified group respectively. Therubric score and global rating score, also the pass rate between two learningmethods showed no significant results (*P* > 0.05). The satisfaction and self-confidence questionnaires got answers ‘agree and strongly agree’ for all questions.

**Conclusion:**

The learning method using modified and classic Peyton Four-StepApproach were equally effective for learning basic skill of endotracheal intubation and mask ventilation for students of the Faculty of Medicine,University of Indonesia. Both methods provided equal students’ satisfaction and self-confidence.

## Introduction

Airway emergency is a life-threatening condition that commonly occurs outside the operating room. The International Observational Study to Understand the Impact and Best Practices of Airway Management in Critically Ill Patients (INTUBE) stated that the most common underlying cause of intubation was respiratory failure, approximately 52,3% [1]. Endotracheal intubation and mask ventilation, as airway management for airway emergency cases, is a basic procedural skill that should be mastered by a general practitioner without supervision in Indonesia based on *Standar Nasional Pendidikan Profesi Dokter Indonesia* (SNPPDI) 2019; therefore, it has to be learned by medical students and practiced directly [2,3]. Endotracheal intubation skill learning is taught to medical students during the Basic Clinical Skills module, which has been proven to effectively improve students’ skills compared to standard training or without training, resulting in better performance in the objective structured clinical examination (OSCE).

However, during the COVID-19 pandemic, offline training was difficult to conduct, and new learning methods such as online learning and distance learning were developed [4]. Government has encouraged institutions to start distance learning or online classes using digital technology, which allows classes while teachers and students are not at the same place [5]. Before the pandemic, the Basic Clinical Skills module adopted Peyton’s Four-Step Approach, which is implemented directly through face-to-face training, yet a new specific learning method during the pandemic has not yet been established. This training method began with skills demonstration and explanation directly from tutors, which were repeated by the students, and direct hands-on by medical students. All the sessions were done offline. A study in the United Kingdom examined the implementation of modified Peyton’s Four-Step Approach using videos for demonstration and online technology, which resulted in satisfactory outcomes and feedback from the students [6]. The demonstration and explanation by tutors were done online. The students watched YouTube videos made by tutors, followed by a session to discuss the skills. The students were asked to make a new video of the skills using a video recorder inside the skill lab room and ended with feedback sessions through Zoom meeting.

Since the end of the pandemic could not be predicted and endotracheal intubation and mask ventilation procedural skill training needed to be performed, a modification of Peyton’s Four-Step Approach was proposed to be implemented using online and distance learning methods. This study aimed to assess the effectiveness of the modified Peyton’s four-step approach, which can be implemented through online training, compared to the classic Peyton’s four-step approach in learning endotracheal intubation and mask ventilation procedural skills.

## Methods

### Study design

This experimental study was conducted in the Faculty of Medicine, specifically in the Skill Lab room training, during the Basic Clinical Skill module for five months, from February to June 2022 with clinical trial registration number NCT05511142. Two hundred and twenty-nine second-year medical students attending the Basic Clinical Skills module of endotracheal intubation and mask ventilation were divided into two groups, group one received face-to-face training using Peyton’s four-step approach (classic group) and group two received modified Peyton’s four-step approach through online or distance learning (modified group). Peyton’s four-step approach was selected through a review and discussion between researchers as the most effective and suitable method for this research compared to others. The assessment was performed by comparing the OSCE end score using the rubric and global rating scores, as well as a satisfactory score from the feedback questionnaire. The scores were used as primary data for this research after obtaining permission from the ethics committee and written informed consent from medical students. Students included in the research must have attended the entire session of endotracheal intubation and the mask ventilation module.

Second-year medical students who had been attending endotracheal intubation and mask ventilation modules or had followed similar training before were excluded. Students who quit or discontinued participation in the module or were not present in one or more training sessions were excluded.

### Subjects’ enrolment

Two hundred forty-two students met the eligibility criteria and were divided into two groups. All students gave consent to participate on and follow the study. Two students from the classic group were excluded because of previous experience with endotracheal intubation and mask ventilation training. Four students from the classic group and seven students from the modified group dropped out because of their absence in any training session. Simple convenience sampling was conducted and the allocation for each group was performed in a random manner by the team of Basic Clinical Skills Module. We made sure that this training was the first endotracheal intubation and mask ventilation training for all students; thus, the basal level of both groups was equal. The classic group of 133 second-year students, divided into a smaller group of 5 to 6 people, received face-to-face learning using classic Peyton’s four-step approach, implementing the COVID-19 preventive health protocol. The modified group of 96 students was also divided into a smaller group of 5 to 6 people, received distance learning through zoom meetings using modified Peyton’s four-step approach.

### Activity

The learning objectives of this new training method were to enable second-year students to perform procedural skills, especially endotracheal intubation and mask ventilation, in the midst of the pandemic COVID-19 which limits offline training. This new approach was discussed among qualified trainers and tutors with more than 10 years of experience to continuously develop a more effective module to be implemented as a learning tool for medical students.

The new approach consisted of four steps throughout the five months of the basic clinical skills modules. All module learning materials, including a checklist of skills, were reviewed and selected by experienced trainers and tutors. After training and testing, each student was graded as ‘pass’ or “fail, respectively.

Step 1: Second-year students watched YouTube videos made by tutors without any comment or discussion.

Step 2: Discussions between second-year students and tutors were conducted. The tutors gave explanations about the video, asked questions, and interacted with the students. Tutors can ask students to follow a video.

Step 3: Second-year students made a new video of endotracheal intubation and mask ventilation procedure with a mannequin and equipment inside the skill lab room using a video recorder owned by the students and uploaded the video to Google Drive. The students were required to be on campus.

Step 4: Tutors and students had a meeting via Zoom to discuss the video made by the students, and the tutors gave feedback to each student.

The old method (the classic approach) is a classic face-to-face learning method. The training session began with endotracheal intubation and mask ventilation demonstrations without explanation by the tutor, followed by another demonstration with explanation by the tutor and demonstration by the tutor and explanation by medical students, a discussion session, and direct hands-on by medical students, and ended by giving feedback to each other.

### Student assessment

After the completion of the module, all participants were performing OSCE, as the part of the curriculum for medical students at the institution to assess the ability to do procedural skills, including mask ventilation and endotracheal intubation skills. The examiners were not part of the research team. The skills were assess using the national standard assessment for OSCE for the specific skills. We used the intubation and mask ventilation checklist as the tools for assessment (Supplementary Table S1). The assessment contain criteria as follows: Skills performance (rubric) and Global rating scale. Both criteria contribute to end-result of the assessment. Rubric score is a quantitative score which sums the points of several aspects accomplished by the student in performing the intubation and mask ventilation procedure. Each step of the rubric was assessed using scale 1 to 4; 1: initial, 2: developing, 3: proficient, 4: advanced. The global rating scale is a qualitative score, which reflects the examiner’s opinion once the students have completed the item score list. It was assessed using scale ‘superior’, ‘pass’, ‘borderline’, and ‘fail’. The researchers asked permission from the program director to analyse the participants score for mask ventilation and endotracheal intubation stations. Finally, a satisfactory and self-confidence survey was administered to second-year student to ascertain their view on using the new approach and to obtain feedback on the module (Supplementary Table S2).

### Statistical analysis

Data were analyzed using the Statistical Package for the Social Sciences or SPSS® software (version 20.0; SPSS®, Inc., Chicago, IL, USA). The difference in the OSCE scores between the two groups was analyzed. The Kolmogorov – Smirnov test was performed to test for normal distribution. Numerical data with normal distribution were analyzed using an unpaired t-test to compare the mean OSCE score in each group. Numerical data with abnormal distributions were analyzed using the Mann-Whitney U test. Categorical data were analyzed using the chi-squared test. Statistical significance was set at *P* < 0.05.

The questions on the feedback form were presented on a five-point Likert scale. The results of the feedback questionnaire were analyzed using the Mann – Whitney test or unpaired categorical comparative with the 2×K table.

### Ethical statement

Sampling was performed after obtaining approval from the Ethics Committee of the Faculty of Medicine, University of Indonesia (No. 758/UN2.F1/ETIK/PM.00.02/2021) on NaN Invalid Date NaN.

## Results

Two hundred and twenty-nine second-year medical students from the Faculty of Medicine Universitas Indonesia participated in this study; 133 students received training with the classic Peyton’s four-step approach and 96 students with the modified Peyton’s four-step approach. All students were currently in their second year, and the detailed demographic data are presented in Table 1.

**Table 1.** Demographic data.

		Method		
Variable		Classic (*n* = 133)	Modified (*n* = 96)	P value
Gender, *n (%)*	Male	54 (40,6)	39 (40,6)	0,997^a^
	Female	79 (59,4)	57 (59,4)	
Class, *n (%)*	Regular	106 (79,7)	71 (74,0)	0,306^a^
	International	27 (20,3)	25 (26,0)	
Age, *mean (SD)*		19,53 (0,043)	19,46 (0,051)	0,312^b^

^a^Chi-square test. ^b^T-test.

Abnormal data distribution of rubric scores from the two groups was analyzed using an unpaired t-test, with P = 0,936 (Table 2). The difference in rubric score between the two groups was statistically insignificant, with a median value 21,2. Global rating scores from the two groups were analyzed using the Fisher test because more than 20% of the cells had an expected score of less than five, the chi-square test could not be used. Table 3 shows that there was no significant association between the two learning methods and the global rating score.

**Table 2.** Rubric score.

	Method	N	Median	IqR	P value
Rubric score	Classic	133	21,20	3,55 (19,30–22,85)	0,936
	Modified	96	21,20	2,43 (20,20–22,63)	

T test. N, total; IqR, *interquartile range*.

**Table 3.** Global rating score.

		Method		
		Classic, *n (%)* (*n* = 133)	Modified, *n (%)* (*n* = 96)	P value
Global rating	Superior	27 (20,3)	18 (18,8)	0,112
	Pass	82 (61,7)	57 (59,4)	
	Borderline	22 (16,5)	16 (16,7)	
	Fail	2 (1,5)	5 (5,2)	

Fisher test.

### Pass rate of endotracheal intubation and mask ventilation procedural skill

Passing grade was calculated as the passing criteria for all students participating in endotracheal intubation and mask ventilation procedural skill training. The passing grade was determined with the borderline regression method on the rubric score using the borderline score of the global rating score.

**Figure 1.**
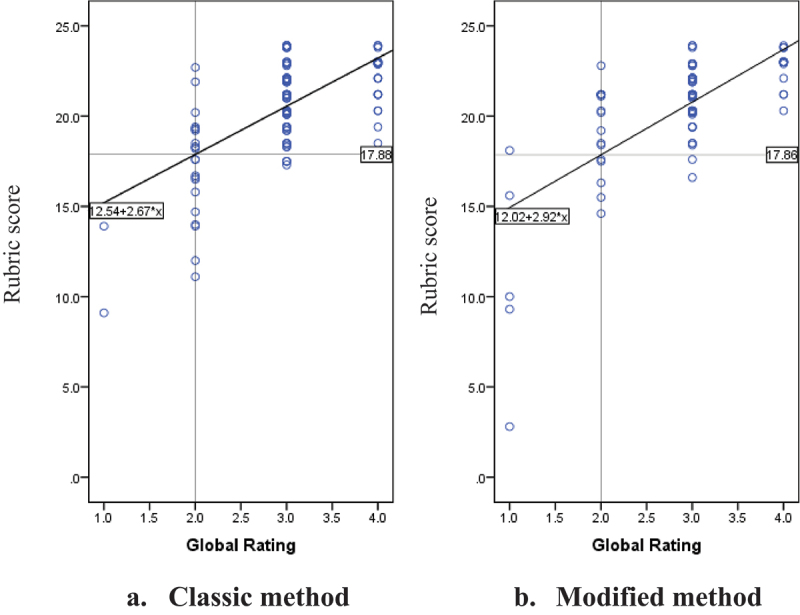
Passing grade of endotracheal intubation and mask ventilation procedural skill using borderline regression method.

The passing grades for the classic and modified group were 17,88 and 17,86, respectively (Figure 1). Both groups showed a strong correlation between the global rating and rubric scores, with R^2^ value of 0,413 and 0,483 for the classic and modified groups, respectively. Based on the passing grade, 87,2% students from the classic group and 88,5% from the modified group passed the training (Table 4). There was no significant difference between the training method and pass rate. Moreover, the difference between pass and fail rates in both groups was 1,3% and 1,3%, respectively. It can be concluded that there is no significant difference in the pass rate between the two methods in terms of the effectiveness of the training method.

**Table 4.** Pass rate of endotracheal intubation and mask ventilation procedural skill training based on passing grade score.

		Pass, *n (%)*	Fail, *n (%)*	P value
Method	Classic	116 (87,2)	17 (12,8)	0,763
	Modified	85 (88,5)	11 (11,5)	

*Chi square test*.

### Feedback questionnaire

All questions analyzing satisfactory feedback from participants were analyzed and resulted in *P* > 0.05 for both groups (Supplementary Table S2). Therefore, there was no significant difference in satisfaction or self-confidence between the two groups.

## Discussion

### Effectiveness and assessment method

We found that the effectiveness of the classic Peyton’s four-step approach and modified Peyton’s four-step approach for endotracheal intubation and mask ventilation training was comparable without significant differences. We compared effectiveness based on learning achievement and examined objectively using scores obtained from the Objective Structured Clinical Examination (OSCE) [2,7]. OSCE could assess at the level of *‘shows’* and *‘shows how’* in Miller triangle hierarchy, which were important to evaluate physicians’ clinical skills. Simulation is the cornerstone of basic clinical skills learning. Laschinger et al. showed that simulation-based learning has been effective in boosting medical students’ self-confidence and satisfaction and has been proven to help prepare themselves in real situations [8]. Khorashad et al. also demonstrated the comprehensiveness of the OSCE and its superiority in assessing practical skills, which could not be evaluated using writing tests [9].

The rubric score was determined by assessing participants’ performance following all the steps based on a checklist made by the examiners, where the global rating score evaluated overall performance. The rubric and global rating scores supported the OSCE to become structured and consistent to be used by every single tutor, with high validity and reliability, and considered as the benchmark of performance-based clinical assessment in medical education [10]. Previous studies have showed a higher inter-reliability and validity for global ratings than checklist scores [11,12]. A multi-centered study conducted in Indonesia showed that OSCE using rubric and global rating scale in each station had good validity and reliability; thus, it was feasible and acceptable to be implemented in large-scale and multi-site for national competency examination in Indonesia [13]. Global rating scale (GRS) reflects examiners’ expert opinion towards overall skill performance, which is converted into four levels of scales: fail, borderline, pass, and superior. Compared to the rubric score, the GRS showed higher internal consistency and inter-rater reliability, thus providing further evidence for the reliability of subjective examiner ratings [11,14].

Gradl-Dietsch et al. stated that Peyton’s four-step approach is more effective as clinical skill training learning method compared to traditional learning method, such as ‘see one, do one.’ [15,16] Peyton’s four-step approach comprises four steps: demonstration, deconstruction, comprehension, and performance/execution [15]. Learning process on this method uses simulation-based medical education (SBME), adopting ‘mistake forgiving’ principle which allows students and tutors to discuss and explore more detail about the skill [17,18].

The ratio between the number of tutors and students in a group could also affect learning effectiveness, as stated by Giacomino et al. Peyton’s four-step approach has been proven to be more effective if implemented in a small ratio of tutor-students; therefore, a group of 5 to 6 students was considered to be ideal, considering the total number of students participating in the basic clinical skill module. Lund et al. implemented the SBME model in clinical skill training, offered a promising outcome, and showed the superiority of the Peyton approach compared to the traditional bed-side teaching method. However, its long-term impact is still needed to be further analyzed [19]. The learning environment and greater chance of observing, discussing, and trying to perform the skill were also associated with higher achievement of learning objectives. Repetition plays a key role in achieving learning objectives, specifically information retention. Spaced repetition is considered to be the best way to process information in long-term memory. The more the repetition and review process implemented, the longer the duration of the information retained in the human memory system. In this research, the OSCE was conducted 2–3 months after the training session; however, the students still performed well, showing good retention of information understood during training [19,20].

Modified Peyton’s four-step approach was implemented as an adaptation to the pandemic COVID-19, where the classic method was impossible to perform. It used video to replace direct demonstration; although it was still not an ideal way, it was considered to be the best solution to overcome the limitations of the pandemic [6]. Videos have been important part of educational learning during the pandemic [21]. Seifert et al. compared the effectiveness of video-based learning using Peyton’s four-step approach and a traditional demonstration method and showed superiority in the Peyton approach [22].

### Clinical skill assessment

Based on our findings, the classic and modified Peyton’s four-step approach were as effective in achieving learning objectives because every crucial step had still been done in both methods. The modified Peyton’s four-step approach required a longer learning duration, up to 4–5 days, compared to the classic method, which could be done in just a day. In modified approach, there were more steps which could not be performed in one day. Eventually, it favored spaced learning method, which allowed spacing intervals and spaced repetition in practicing the skill. Chugh et al. showed that spaced education with extended time learning statistically improved knowledge retention among medical student [23]. In *demonstrate* step, the classic group observed the simulation directly, whereas the modified group was observed through a video. In *deconstruction and comprehension* steps, there was no significant difference, where students were asked to perform the procedure and explain each step. In *performance/execution* step, the classic group performed directly in front of the tutor, whereas the modified group only had the chance to discuss and clarify their understanding via online meetings. However, Nikendei et al. stated that the most crucial step is *comprehension*, which is performed in the same manner in both groups [24]. Therefore, even though the modified method of direct hands-on or practice could not be performed, the effectiveness is still the same.

Zalat et al. showed positive feedback from tutors and students regarding the implementation of online learning methods. Online learning methods could improve tutors’ learning experiences and education skills, encouraging further exploration of how to facilitate student learning in different ways. The students also found the learning method convenient with a high rate of acceptance [25]. It supported our findings, in which both students and tutors felt satisfied with the modified method, realizing that a change must be made during the COVID-19 pandemic (Supplementary Table S2).

This study has several limitations. Medical students in the modified group often attended the online class at night, which could have affected their stamina and ability to focus on the training and made the session less effective. Several mannequins provided by the institution were inappropriate because of the damage. Medical students performed direct laryngoscopy on the video; therefore, the tutors found it difficult to assess and give feedback, as direct observation of the process was impossible. Finally, the OSCE was done 2–3 months after the training, which was considered quite long; thus, the direct effect could not be assessed.

## Conclusion

The modified Peyton’s four-step approach is as effective as the classic Peyton’s four-step approach for endotracheal intubation and mask ventilation procedural skill training for medical students. There was no significant difference in rubric and global rating scores between the two groups. Satisfaction and self-confidence levels obtained from the feedback form were also comparable between the two groups. Therefore, the modified Peyton’s four-step approach can be used when face-to-face training cannot be performed.

## Supplementary Material

Supplemental Material
