# Supplementary material for: Effectiveness of endotracheal intubation and mask ventilation procedural skills training on second-year student using modified Peyton’s Four-Step approach during COVID-19 pandemic
Source: Med Educ Online. 2023 Sep 7;28(1):2256540. doi: 10.1080/10872981.2023.2256540 (PMC10486285; doi:10.1080/10872981.2023.2256540)
Supplement: Supplemental Material [file ZMEO_A_2256540_SM0199.docx]

**Supplementary Table 1.**

**Intubation and Mask Ventilation Checklist**

| **No** | **Skill** |  |
| --- | --- | --- |
|  | **Preparation** |  |
| 1 | Check the completeness of equipment |  |
| 2 | Use personal protective equipment (PPE), such as gloves and goggle |  |
| 3 | Explain the procedure to the patients if awake or their family if unconscious, and asking for written consent |  |
| 4 | Ensure the airway is open and clear |  |
| 5 | Ensure an adequate oxygenation and ventilation |  |
| 6 | Ensure an adequate IV access |  |
| 7 | Prepare and set the monitor |  |
| 8 | Prepare the endotracheal tube  Check endotracheal cuff for leaks  Spread lubricant on the stylet and insert the stylet inside the endotracheal tube  Spread lubricant from the cuff to the tip of tube |  |
| 9 | Prepare the laryngoscope  Prepare the blade  Ensure the light is on |  |
| 10 | Place a pillow or cloth under the occipital part of head if there is no suspected cervical injury |  |
| 11 | Spray topical analgesia to the oropharynx (xylocaine spray : 1 puff = 10 mg, given based on the dose) |  |
| 12 | Preoxygenate the patient with 100% oxygen 2-3 minutes |  |
| 13 | If needed, give sedation, analgesia, and muscle relaxant |  |
|  | **Technique** |  |
| 1 | Operator stands behind patients’ head. The bed is positioned without elevation |  |
| 2 | Hold the laryngoscope with left hand, ensure the patient is already unconscious |  |
| 3 | Ask the assistant to press the cricoid and hold on still until the tube is correctly inserted |  |
| 4 | Open the mouth with cross finger technique, crossing the index finger and thumb of the operator's nondominant hand like the blades of a scissor. While the index finger rests on the maxillary teeth, the thumb is placed over the mandibular teeth and the two crossed. |  |
| 5 | Slowly insert the blade into the right side of the patient’s mouth, insert until it reaches the base of the tongue |  |
| 6 | push the tongue to the left |  |
| 7 | The tip of the curved blade should be placed in front of the epiglottis in the valecula. The tip of the straight blade should be placed under the epiglottis. |  |
| 8 | Visualize the vocal cords and glottis opening |  |
| 9 | Gently insert the ETT along the right side of the mouth under direct visualization of the vocal cords, grasp the ETT tube with the right (dominant) hand |  |
| 10 | Remove the stylet and laryngoscope while the right hand still holds the tube |  |
| 11 | Inflate the balloon cuff |  |
| 12 | Assess for proper placement of ETT:  Attach the bag-valve-mask and end tidal CO2 monitor  Fogging in ETT  Bilateral breath sounds and symmetric chest movement |  |
| 13 | Fixate the secure the ETT with tape at the number written on the ETT, at lips level |  |

**Supplementary Table 2**

**Feedback Questionnaire**

| **Question** | **Indicator** | **Classic, *mean (grade)*** | **Modified, *mean (grade)*** |
| --- | --- | --- | --- |
| Learning process | The training can improve my problem-solving skill | 4,39 (SA) | 4,29 (SA) |
|  | The training can help me work in a team | 4,36 (SA) | 4,44 (SA) |
|  | The training improves my intubation skill | 4,54 (SA) | 4,65 (SA) |
|  | The training improves my ventilation mask skill | 4,53 (SA) | 4,56 (SA) |
|  | The training can improve my confidence if I have to intubate patients | 4,31 (SA) | 4,36 (SA) |
|  | The training can improve my judgement in deciding when intubation is needed | 4,35 (SA) | 4,21 (SA) |
|  | The training is exciting | 4,56 (SA) | 4,46 (SA) |
|  | The training improves my understanding during exam | 4,51 (SA) | 4,60 (SA) |
|  | I am satisfied with the training | 4,46 (SA) | 4,50 (SA) |
| Trainers | The trainers help me learning the skill | 4,54 (SA) | 4,56 (SA) |
|  | The trainers teach and demonstrate intubation and ventilation mask skill effectively | 4,52 (SA) | 4,50 (SA) |
|  | There is enough time to practice and discuss the material | 4,37 (SA) | 4,45 (SA) |
| Learning material | The learning objective is clear | 4,54 (SA) | 4,56 (SA) |
|  | The learning tools help the learning process | 4,49 (SA) | 4,46 (SA) |
|  | The time given is enough to practice | 4,18 (A) | 4,31 (SA) |
|  | The mannequin is sufficient for practice | 4,14 (A) | 4,11 (A) |
|  | The equipment provided for practice is sufficient | 4,30 (SA) | 4,26 (SA) |

SA = Strongly Agree (4,2 – 5)

A = Agree (3,4 – 4,19)

N = Neutral (2,6 – 3,39)

D = Disagree (1,8 – 2,59)

SD = Strongly Disagree (1 – 1,79)
